# Catastrophic Health Impacts of Spiraling Climate Change: How Certain Can We Be About Their Magnitudes?

**DOI:** 10.3389/fpubh.2020.584721

**Published:** 2020-11-26

**Authors:** Zaid Chalabi, Anna M. Foss

**Affiliations:** ^1^Bartlett School of Environment, Energy and Resources, University College London, London, United Kingdom; ^2^Faculty of Public Health and Policy, London School of Hygiene and Tropical Medicine, London, United Kingdom

**Keywords:** human health impacts, catastrophic health risks, climate change, mathematical modeling, handling uncertainty

## Abstract

Recently, there has been a strong interest in the climate emergency and the human health impacts of climate change. Although estimates have been quoted, the modeling methods used have either been simplistic or opaque, making it difficult for policy makers to have confidence in these estimates. Providing central estimates of health impacts, without any quantification of their uncertainty, is deficient because such an approach does not acknowledge the inherent uncertainty in extreme environmental exposures associated with spiraling climate change and related health impacts. Furthermore, presenting only the uncertainty bounds around central estimates, without information on how the uncertainty in each of the model parameters and assumptions contribute to the total uncertainty, is insufficient because this approach hides those parameters and assumptions which contribute most to the total uncertainty. We propose a framework for calculating the catastrophic human health impacts of spiraling climate change and the associated uncertainties. Our framework comprises three building blocks: (A) a climate model to simulate the environmental exposure extremes of spiraling climate change; (B) a health impact model which estimates the health burdens of the extremes of environmental exposures; and (C) an analytical mathematical method which characterizes the uncertainty in (A) and (B), propagates the uncertainty in-between and through these models, and attributes the proportion of uncertainty in the health outcomes to model assumptions and parameter values. Once applied, our framework can be of significant value to policy makers because it handles uncertainty transparently while taking into account the complex interactions between climate and human health.

## Introduction

In May 2019, the UK Parliament declared a climate change emergency (1). A year on, during the ongoing COVID-19 crisis and associated lockdown measures, there has been strong advocacy pressure to build a green recovery plan that addresses the climate emergency and related human health effects (2). However, there is often resistance to implementing the global transformational changes required in society to avoid spiraling climate change because of their perceived negative impact on the economic development of the current human generation in favor of future generations. It is important, therefore, to provide policy makers with strong evidence on the consequences of inaction, particularly in relation to the catastrophic human health impacts of spiraling climate change.

There are several pathways from spiraling climate change to health (3). These include heatwaves, lack of water availability, loss of crop yields, increase in communicable and vector-borne diseases, social and political upheaval, migration and conflict. These impacts are not necessarily instantaneous but can accrue over different time scales and affect different populations unequally depending on their geographical locations. For example, floods can have short-term and long-term health effects. Short-term effects include water shortages, disruption to food supplies, livelihood and incomes; whereas long-term effects include soil and water contamination as well as mental health issues (3).

Modeling the health impacts of spiraling climate change is a very complex problem because it entails interlinking between different models of climate, environment and human health. Various estimates have been provided for the best/worst case climate change scenarios (4). Yet, how convincing are these estimates to policy makers?

We argue that many of these impact calculations are either opaque or simplistic. Providing central estimates of human health impacts without any quantification of their uncertainty is deficient because such an approach does not acknowledge the inherent uncertainty in extreme environmental exposures associated with catastrophic climate change and their health impacts. Furthermore, presenting only central estimates of impacts can give unwarranted confidence in the model outputs. It is imperative, therefore, that the central estimates are provided within bounds to reflect the level of uncertainty in these predictions. It is also important that the total uncertainty in any impact estimate is decomposed in order to determine the contribution of uncertainty in each of the main model parameters and model assumptions to the total uncertainty in the model output. Instead, we require a systematic and transparent approach to modeling which allows as much mathematical scrutiny as possible.

The importance of quantifying the uncertainty in the direct and indirect impacts of climate change on health and wellbeing has been recognized for some time (5, 6). Various approaches have been proposed for this purpose. However, the level of uncertainty in the health impacts of spiraling or catastrophic climate change far exceeds that of the impacts of current climate change (3). This is due to the large uncertainty in the climate model at the climate tipping points, the induced levels of the environmental exposures and the response of humans to these exposures (for which there is no evidence because the climate has not yet reached its tipping points). This escalating uncertainty, associated with spiraling climate change, requires careful attention. We believe that our proposed framework, described below, holds promise in addressing this escalating uncertainty.

When calculating the human health impacts of spiraling climate change, potential methodological approaches may span the full range of the modeling spectrum, from basic analytical spreadsheets to large-scale computer simulation models. The former extreme gives us transparent models that are easy to use and discuss with policy makers but may be over simplistic, while the latter extreme can incorporate useful all-encompassing complexity, but such models may be opaque in terms of being hard to communicate effectively to policy makers. We believe that a hybrid approach, that appropriately balances the need for transparency (e.g., in how uncertainty is handled) with a suitable level of complexity (e.g., to capture the complex interplay between spiraling climate change, environmental exposures and their related health burdens), should be the basis of any model used to calculate catastrophic human health impacts due to spiraling climate change. For this purpose, we advocate the use of standalone state-of-the art climate and human health impact models, used in conjunction with analytical mathematical approaches which link these two sets of models. We assert that only an analytical mathematical approach is able forensically to thread the connections between the various models and which has, at its base, a method for uncertainty quantification, propagation and attribution, through the models. We outline one such potential framework below.

The purpose of our framework is to provide an approach to handle uncertainty in the nexus of spiraling climate change and health. We advocate the use of rigorous mathematical methods to quantify and propagate the uncertainty in the climate and health models. Our aim is not to prescribe specific climate and health models *per se* but to promote the use of mathematical methods to deal with the uncertainty in a series of linked models. Current approaches for handling uncertainty treat the uncertainty in each model separately without linking the uncertainties in the series of models from the climate model to the health model via environmental exposures. This is a weakness of many environmental health models because, ideally, the uncertainty should be propagated coherently throughout the series of models (5, 7). Quantifying the health impacts of spiraling climate change is even more challenging because of the nature of the large uncertainties in the climate-health linkages. We propose appropriate mathematical methods for quantifying the overall uncertainty and attributing it to the different sources.

## Framework

**Figure 1:**
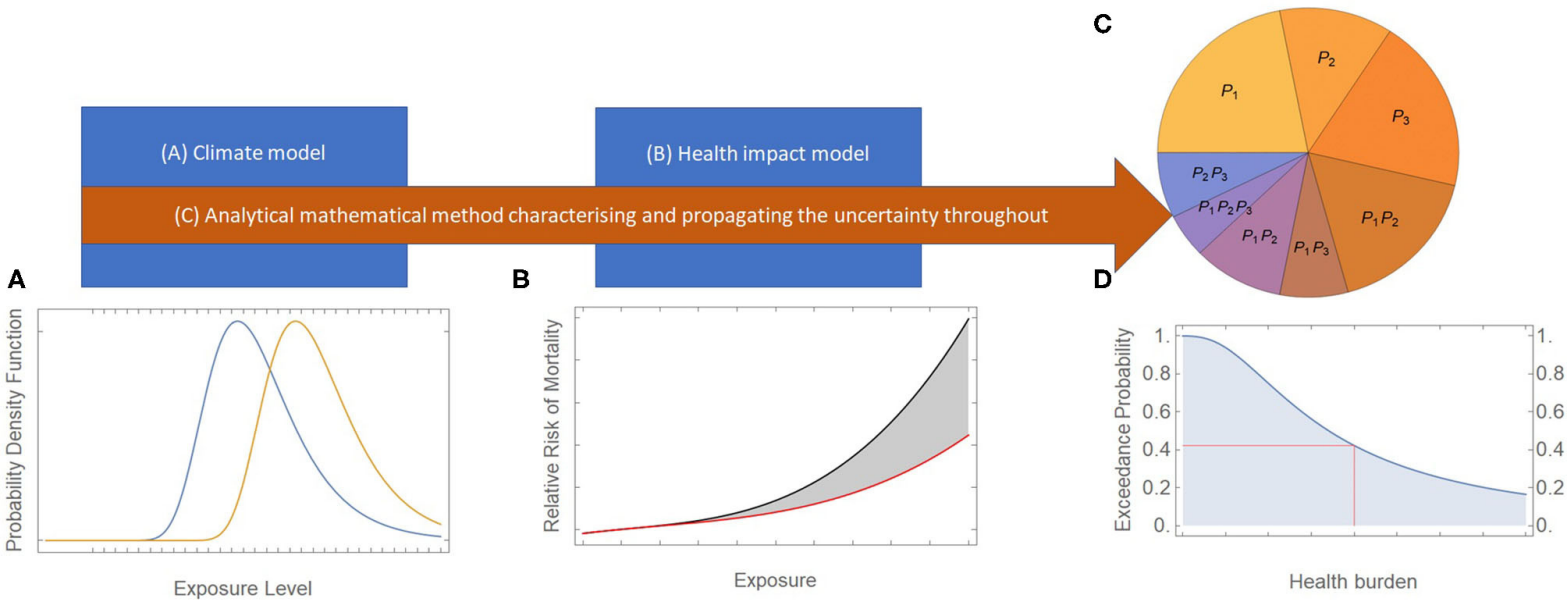
Our framework connecting building blocks (A), (B), and (C) to the final outcome, namely the catastrophic human health burden of spiraling climate change (and the associated uncertainty in this estimate). **(A)** provides an example of the probability density function of the maximum (extreme) of an environmental exposure (e.g., temperature) over two non-overlapping discrete time points. **(B)** is an example of an exposure-response relationship, showing the effect of an environmental exposure on human health (mortality). **(C)** considers a hypothetical 3-parameter model (*P*_1_, *P*_2_, *P*_3_), where each slice of the pie chart illustrates the hypothetical percentage contribution toward the total uncertainty. **(D)** is an illustrative example of the exceedance probability of catastrophic human health burden.

Our framework, depicted in Figure 1, comprises three building blocks:

- a climate model to simulate the environmental exposure extremes of spiraling climate change;
- a human health impact model which estimates the health burdens of the extremes of environmental exposure; and
- an analytical mathematical method which characterizes the uncertainty in each model from (A) and (B), propagates the uncertainty in-between and through these models, and attributes the proportion of uncertainty in the health outcomes to model assumptions and parameter values.

Before describing these building blocks in more detail, we must first consider our desired final outcome, namely the catastrophic human health burden of spiraling climate change (and the associated uncertainty in this estimate). Calculating such burdens requires knowledge of the likelihood of the events causing them and the extent of the damage that they could cause. Historically, there has always been an interest in calculating the likelihood and impacts of all types of catastrophic events ranging from physical, astronomical and biological events (e.g., tsunami, asteroids, plagues, pandemics), to social and political events (e.g., nuclear war, social collapse) (8). The impact of any extreme event can be captured by determining the exceedance probability of the “damage” (in our case, “human health burden”), caused by the event (or a series/combination of events, as in our case of spiraling climate change). The exceedance probability gives the probability that a certain burden is exceeded (Figure 1D).

In the illustrative schematic given in Figure 1D, the x-axis is the human health burden (or its logarithm to accommodate both small and extremely large-scale impacts) and the y-axis is the exceedance probability. Any point on the curve gives the probability that the incurred burden is greater than or equal to that associated with the point. More specifically, the two lines perpendicular to the axes from a point on the curve give the probability (where the line perpendicular to the y-axis cuts the y-axis) that the burden exceeds that associated with the point (where the line perpendicular to the x-axis cuts the x-axis). The curve starts at probability 1 (corresponding to the certainty that the health burden exceeds zero or a minimal level) and rapidly declines at first, for low levels of health burden, before more gradually decreasing for high and extreme levels of health burden.

Figure 1D simulates the exceedance probability of an event characterized by an extreme value distribution (i.e., “fat-tailed” distribution) where the probability of catastrophic damage is not small and can be significant. This type of characterization of extreme risks originated initially in engineering to quantify the catastrophic failures of structures (9) and has recently been applied to quantify the human health effects of extreme weather events (10, 11). The premise of Figure 1D is that spiraling climate change will induce very large-scale changes in the extremes of environmental exposures (Figure 1A) which will, in turn, impact human health (Figure 1B).

Since we are interested in the extremes of environmental exposures, such as extreme temperatures, these are best represented by time-varying (non-stationary) Generalized Extreme Value (GEV) distributions. A GEV describes the probability distribution of an extreme variable (e.g., a minimum or a maximum). Figure 1A shows schematically the hypothetical change in a time-varying GEV probability density function (PDF^1^) of the maximum of an environmental exposure as it changes over time with spiraling climate change. In this figure, it is assumed that the environmental exposure increases dramatically over time. The area under the PDF between any two points gives the probability that the exposure lies between the two points. It is hypothesized in Figure 1A that the distribution of the maximum of an environmental exposure shifts significantly to the right with spiraling climate change, meaning that the probability of very high exposures is greater at a later time point (with the PDF of the earlier time point shown in blue and that of the later time point in orange). Estimates of these distributions and how they change over time can be determined from General Circulation Models (12), the large-scale climate models referred to in building block (A) above.

Moving onto building block (B), the change in the distribution of an environmental exposure impacts human health, which can be calculated via an exposure-response relationship. Figure 1B shows schematically such a relationship at high exposure levels, which is assumed here to be nonlinear. The y-axis is the human health burden, characterized in this example by the relative risk of mortality, and the x-axis is the exposure level. In the case of some exposures, such as temperatures, there is ample epidemiological evidence to quantify exposure-response relationships (13), while data is lacking for some others. Because of the uncertainty in the exposure-response relationship at very high exposures, this relationship is represented by an uncertainty band in Figure 1B. The uncertainty in the relationship is captured in the area between the lower and upper curves. The lower and upper curve could represent, for example, the 95% confidence interval around the central estimate of the relationship.

**Figure 2:**
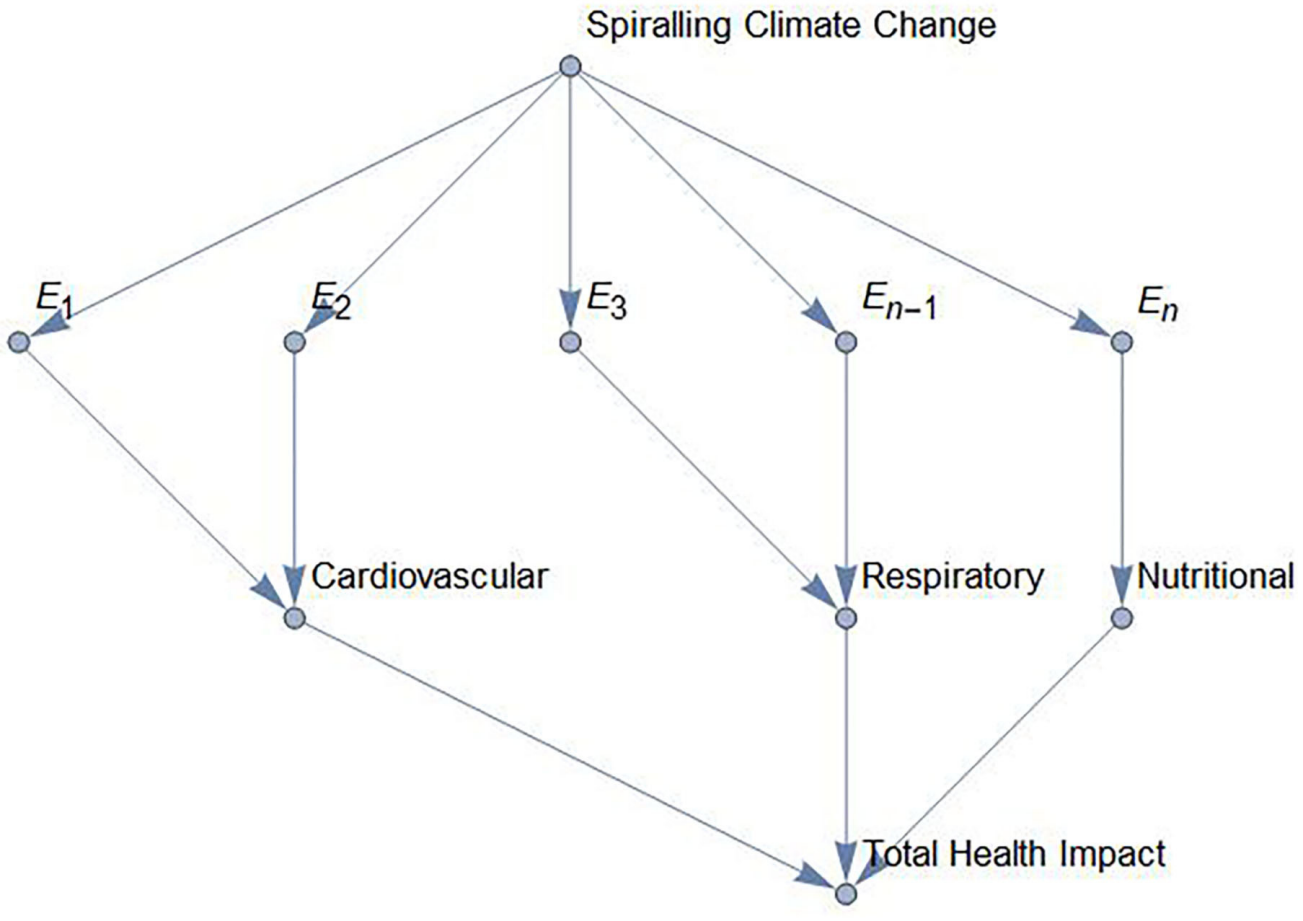
Causal relationships between spiraling climate change and human health via *n* environmental exposures, *E*_1_ *to* *E*_*n*_.

Figure 2 shows schematically the causal pathways between climate change and human health via several environmental exposures (*E*_1_ to *E*_*n*_) such as extreme temperatures, pollution and rainfall. The figure shows mortality and morbidity impacts. If these impacts are expressed by a common health metric such as Quality Adjusted Life Years (QALYs) or Disability adjusted Life Years (DALYs), the total health impact is given by the sum of the mortality and morbidity impacts. For example, there is ample epidemiological evidence on the health effects of extreme hot temperatures on mortality and morbidity including cardiovascular, respiratory and renal diseases (14).

In the case of extreme heat, the nature of the curvature of the exposure-response relationship at extreme hot temperatures would depend on the adaptation potential of the affected population. For example, where there is provision of air conditioning (which depends on the socio-economic status of the population), the curvature could be less steep.

Since there are multiple environmental exposures (as illustrated in Figure 2), it is important to note that these can be independent, linearly related (i.e., correlated) or nonlinearly related. If the environmental exposures are independent and occur far apart in time, their human health impacts (*H*) are additive. However, the human health impacts can also be sub-additive or supra-additive. Sub-additivity means that, if changes in two environmental exposures (*E*_*i*_, *E*_*j*_) occur simultaneously, then their combined health impact is less than or equal to the sum of the impacts of the two exposures if they had instead occurred far apart in time [i.e., *H*(*E*_*i*_ + *E*_*j*_) ≤ *H*(*E*_*i*_) + *H*(*E*_*j*_)]. Conversely, supra-additivity means that, if changes in the two exposures occur simultaneously their impact is greater than or equal to the sum of their health impacts if the exposures had instead occurred far apart in time [i.e., *H*(*E*_*i*_ + *E*_*j*_) ≥ *H*(*E*_*i*_) + *H*(*E*_*j*_)]. To illustrate these hypothetical concepts, consider first a heatwave and an extreme episode of air pollution. If these events occur far apart in time, the total health impact is expected to be additive because there is no interaction between the two impacts. On the other hand, if the two events occur simultaneously, then the total health impact is likely to be supra-additive because health is already compromised by one of the extreme events. Finally, sub-additivity is likely to occur if, for example, there is physiological adaptation to a repeated exposure over a prolonged period, e.g., if the human body copes better over time with repeated exposure to extreme heat. Most health impact frameworks, such as Comparative Risk Assessment (CRA) in the global burden of disease calculations, assume that the health impacts of different environmental exposures are additive (15). However, it has been recognized that impacts of different exposures are not necessarily additive, for example, in relation to environmental pollutants (16).

As Figure 1 illustrates, the method for characterizing and propagating uncertainty in block (C) starts with the uncertainties in exposures in block (A) which are then propagated through to those of the exposure-response relationships in block (B) to quantify the exceedance probability of the human health impacts (quantifying Figure 1D). This process also needs to take into consideration any assumptions or findings on the additivity/non-additivity characteristics of human health impacts with environmental exposures. The method for block (C) can be carried out computationally through Monte Carlo simulations, analytically through applying the theory of random variables (17), or through a combination of both methods (18). Both mutual information analysis (MIA) (19) and global sensitivity analysis (GSA) (20) have been used previously to quantify the contribution of each source of uncertainty to the total uncertainty in the human health impact predictions (21, 22). MIA between a model outcome **Y** and a model parameter ***P*** is the amount of information that ***Y*** and ***P*** share or, equivalently, the amount of uncertainty that is reduced in ***Y*** if the uncertainty in ***P*** is eliminated. GSA decomposes the total uncertainty in a model outcome ***Y*** into the uncertainty of the model's parameters ***P***_**1**_**…*P***_***m***_ and their interactions. For example, consider a three-parameter model. For illustrative purposes, the three parameters could be the relative risk of temperature-related mortality (***P***_**1**_**)**, heatwave duration (***P***_**2**_) and heatwave intensity (***P***_**3**_). As shown in Figure 1C, the total outcome uncertainty could then be decomposed through GSA into the proportion of uncertainty contributed by each parameter separately **(*P***_**1**_, ***P***_**2**_, ***P***_**3**_), two-way interactions between parameters **(*P***_**1**_***P***_**2**_, ***P***_**1**_***P***_**3**_, ***P***_**2**_***P***_**3**_**)** and the three-way interaction between parameters **(*P***_**1**_***P***_**2**_***P***_**3**_**)**. It is just as important to quantify this decomposition of total uncertainty across parameters as it is to estimate the total uncertainty itself.

## Discussion

It is imperative that the potential catastrophic impacts of an inadequate response to climate change are determined with confidence. Our framework above provides a systematic approach for calculating the catastrophic human health impacts of spiraling climate change which we believe is sufficiently transparent to allow for open scrutiny. Each of its components, the climate model, the human health impact model and the analytical mathematical approach for quantifying the uncertainty, stand on their own merits and can be updated or replaced as and when necessary.

We have proposed a framework to provide estimates of the catastrophic human health impacts and the associated uncertainties which, once applied, can be of great value to policy makers. We believe our framework combines three main tenants of importance to policy makers: transparency, acknowledging the complexity of interactions between the environment and human health, and handling uncertainty.

Policy makers are often presented with uncertainty estimates without recourse on how to use them to support decision-making. Because of its forensic approach to dealing with uncertainty, our framework can be linked in a straight-forward manner to a decision analytical framework for handling extreme and catastrophic risks, for example, the Partitioned Multiobjective Risk Method (9) which has been used for health protection planning for extreme weather events and natural disasters (10). Other examples of decision analytical frameworks that our framework can link to include Multi-Criteria Decision Analysis (MCDA) and Expected Value of Perfect Information (EVPI). By providing credible and trustworthy uncertainty bounds on the central estimates of the catastrophic health risks, policy makers can prioritize interventions which are aimed at mitigating these extreme risks, for example by using MCDA (23). By linking our framework to EVPI, the combined framework can inform policy makers on the value of conducting further research into specific unknowns to resolve uncertainties in model predictions. EVPI is widely used to inform health policy makers of the value of added research (24). Beyond this, the framework can be used to compare the findings of different climate and health models to see whether there is agreement across the models on the uncertainty in the health impacts of spiraling climate change.

When operationalised, the usefulness of our framework can be demonstrated against deterministic frameworks by showing the added value of appropriately handling uncertainty in model predictions of the health impacts of spiraling climate change. We argue that ignoring or inadequately handling uncertainty gives a false sense of the accuracy from model predictions and weakens confidence in them.

## Data Availability Statement

The original contributions presented in the study are included in the article, further inquiries can be directed to the corresponding author.

## Author Contributions

ZC led on the intellectual ideas within the piece while AF helped develop these further, especially in terms of the visual conceptualization of the framework and the overall communication of ZC's ideas. ZC wrote the initial draft of the perspective and then both authors jointly revised this iteratively. Both authors contributed to the article and approved the submitted version.

## Conflict of Interest

The authors declare that the research was conducted in the absence of any commercial or financial relationships that could be construed as a potential conflict of interest.

## Footnotes

^1^A PDF of a random variable at a given value gives the probability that the variable is within a differential (very small) range centered around that value.
